# Vaccination Attitudes and Experiences of Medical Doctors in Croatia amid the COVID-19 Pandemic: A Social Roles Conflict?

**DOI:** 10.3390/vaccines10030399

**Published:** 2022-03-05

**Authors:** Maja Miskulin, Aida Mujkic, Ivan Miskulin, Zvjezdana Lovric Makaric, Emma Kovacevic, Ljiljana Pintaric, Zeljko Pavic

**Affiliations:** 1 Faculty of Medicine Osijek, Josip Juraj Strossmayer University of Osijek, 31000 Osijek, Croatia; maja.miskulin@mefos.hr; 2 Andrija Stampar School of Public Health, School of Medicine, University of Zagreb, 10000 Zagreb, Croatia; amujkic@snz.hr; 3 Division for Epidemiology of Communicable Diseases, Croatian Institute of Public Health, 10000 Zagreb, Croatia; zvjezdana.lovric@hzjz.hr; 4 Faculty of Humanities and Social Sciences, Josip Juraj Strossmayer University of Osijek, 31000 Osijek, Croatia; ekovacevic1@ffos.hr (E.K.); lpintaric@ffos.hr (L.P.); zpavic@ffos.hr (Z.P.)

**Keywords:** vaccine hesitancy, communication, healthcare workers, medical doctors, COVID-19, Croatia, focus group, qualitative research

## Abstract

The study aimed to investigate the range of experiences and attitudes of Croatian medical doctors (MDs) related to vaccination and vaccine hesitancy. In January 2021 three asynchronous online focus groups were held using MRQual, a web-based platform, which included 46 MDs from all three levels of the healthcare system in Croatia. NVivo, a qualitative data analysis software package, was used for the thematic analysis of collected data. The participants expressed a high level of support for the Croatian immunization program and vaccines in general. However, some skepticism was expressed regarding new vaccines and the regulatory processes of their approval. A significant number of participants raised concerns over the approval of COVID-19 vaccines, especially given their rapid development. The results also revealed that the process of communication with patients is often based on the very elaborate categorizations of patients based on previous experience, which leads to prioritizing and a communication breakup when dealing with “problematic patients”. MDs find themselves in a delicate situation where a fine balance between time-consuming communication with patients and the demands for maintaining satisfying vaccination uptake is needed. The situation arises from a social roles conflict that is embedded in wider social values and expectations, since communication problems do not arise in the doctor’s office, and therefore cannot be solved without addressing the social forces that cause trust deficiencies. To achieve better immunization results public health leaders need to better understand the social contexts and constraints of MDs vaccine-related behaviors.

## 1. Introduction

Immunization is one of the most successful and cost-effective health interventions and an important pillar of public health. World Health Organization estimates that thanks to immunization around 2.5 million deaths every year are prevented from diseases like diphtheria, tetanus, pertussis, influenza, and measles [1]. The Croatian Mass Immunization Program (MIP) is a mandatory prevention program introduced in 1948 [2], based on the recommendations provided by the Croatian Institute of Public Health [3]. All mandatory vaccinations are offered free of charge and administered by pediatricians practicing at the primary level of healthcare (for pre-school children) and school medicine specialists within county public health institutes (for school-aged children) [4]. As of 2021, the MIP includes vaccination against eleven diseases [5]. In addition to the mandatory vaccinations in Croatia, there is a possibility to get vaccinated against diseases according to the epidemiological indications [6,7]. According to the law on the protection of the population from infectious diseases in Croatia, the vaccinators are medical doctors (MDs) while other healthcare workers (HCWs) can serve as vaccinators only if they are supervised by MDs [8]. Data demonstrate that primary immunization coverage rates of 95% have not been reached for any of the vaccines in the MIP schedule, except for BCG, but the vaccination coverage is rather high, ranging from 89% (Hib revaccination) to 98% (BCG) [9]. According to the Vaccination Report of the Croatian Institute of Public Health, the proportion of the total Croatian population with completed vaccination is 54.94%, while the proportion of the adult population with completed vaccination is 65.48% [10].

Vaccination attitudes of healthcare workers is an interesting and important phenomenon in itself and also one of the crucial factors in successfully implementing vaccination programs [11], given that receiving a recommendation from healthcare providers strongly influences the decisions on vaccination [12]. However, employing a qualitative study, Karafilakis et al. reported that there is some vaccine hesitancy among HCWs in Croatia, Romania, France, and Greece [13]. In addition, quantitative research confirmed that HCWs do express vaccine hesitancy. For instance, Verger et al. determined that 16% to 43% of general practitioners (GPs) in France sometimes or never recommended at least one specific vaccine to their target patients [14]. A systematic review of studies conducted on the topic of HCWs’ vaccine hesitancy concluded that the level of confidence about vaccines’ safety and efficacy appears to be an important factor in this phenomenon [15], which is also confirmed in other studies [16,17,18]. Somewhat surprisingly, the reasons for vaccine hesitancy among HCWs sometimes can even be related to the lack of knowledge about vaccines [19,20]. Therefore, even though it is expected that HCWs show higher levels of vaccine uptake [21], they are still hesitant to a degree. Since vaccination attitudes among HCWs are a highly context-dependent phenomenon [11], they can also be contingent upon specific characteristics of immunization programs and healthcare systems in general. Moreover, additional factors not related to HCWs’ vaccine hesitancy can affect their work on immunization. For instance, increased workload [22], time constraints [23], and the perception of one’s professional role and capacity [24], all influence vaccination commitment among HCWs. However, the social and professional context of HCWs immunization work is significantly less researched when compared to research on HCWs vaccine hesitancy.

It is reasonable to expect that the COVID-19 pandemic should affect vaccination attitudes, but it is difficult to predict in what way and to what extent. Poland [25] reviewed the effects of the 2009–2010 influenza pandemic and concluded that vaccine hesitancy did not alter during the pandemic. He also underlined the role of HCWs as role models [25]. A systematic literature review conducted by Schmid et al. also concluded that reasons for both seasonal and pandemic influenza vaccine hesitancy were similar [26]. However, in the current context of the COVID-19 pandemic, it is interesting to point out that new vaccines were found to be of particular concern because of the perceived insufficient period of testing, i.e., that there are widespread concerns about their efficiency and safety. Thus, there are some indications that the COVID-19 vaccination coverage rates may be partly the consequence of the various (semi)compulsory epidemiological measures, rather than generally positive vaccination attitudes [27]. Sallam summarized worldwide COVID-19 acceptance rates among HCWs and concluded that three of the eight available survey studies reported rates below 60%, whereas the highest reported rate amounted to only 78.1% [28]. A study conducted by Dror et al. suggests that HCWs involved in treating COVID-19 patients were more likely to self-report the future acceptance of COVID-19 vaccination [29]. A cross-sectional study of vaccine hesitancy regarding COVID-19 vaccination amongst HCWs in Turkey revealed that 68.6% of them were willing to get the COVID-19 vaccine when possible [30]. Among vaccine-hesitant HCWs, two main reasons for vaccine hesitancy appeared: lack of trust regarding the new vaccines and possible side-effects of the vaccination. However, a cross-sectional study conducted among nurses in Hong Kong aimed to determine the impact of the COVID-19 pandemic on influenza vaccination acceptance revealed that the change happened more often in positive than in the negative direction [31], while the same conclusion also follows from the recent repeated cross-sectional study on Italian HCWs [32].

Bearing in mind all the above-mentioned, this study intended to investigate the range of attitudes and experiences of Croatian MDs about the vaccination and vaccine hesitancy in general and, in particular, in Croatia, as well as to evaluate possible changes brought about the COVID-19 pandemic. Consequently, given the broad scope of the topic, the study was focused on four issues: (1) general vaccine hesitancy, if any, and its causes among Croatian MDs, (2) the experiences related to the compulsory Croatian MIP, (3) the experiences of workplace expectations and communication with patients, and (4) the COVID-19 context and its potential short-term and long-term impacts on vaccine hesitancy. Therefore, besides probing into the general vaccination attitudes, we aimed to explore whether specific workplace experiences and mandated social roles can constitute barriers that can potentially decrease vaccine uptakes in the current and future pandemics. With that in mind, we conducted a qualitative study aimed at discovering a multiplicity of medical doctors’ opinions and experiences related to vaccination.

## 2. Materials and Methods

The use of online focus groups in research can be divided into asynchronous focus groups in which participation happens at a different time for every participant and synchronous focus groups where participation is in real-time, e.g., video calls or chats [33,34]. In circumstances where distance is preferred due to the global pandemic, it seems that asynchronous online focus groups represent a suitable method that ensures adequate participation and also participants’ diversity in terms of geography [35,36]. Conducting online focus groups seems to be convenient for research in the area of public health, especially when budget research is limited [29], or participants are busy professionals such as doctors and patients who are limited in their availability to fully participate due to circumstances such as working overload [37].

Being cognizant of that, in this study, we present the results of the online asynchronous focus groups conducted with Croatian MDs during January 2021. When selecting the participants, we ensured that MDs from all levels of the healthcare system in Croatia (primary, secondary, and tertiary) were included to gain different perspectives and opinions about the investigated topic. The participants were selected using of a snowball sampling strategy, wherein a purposive decisions were made in order to achieve diversity according to gender and age. Three focus groups (FG1, FG2, and FG3) with a total of 46 participants were held and the characteristics of each group are shown in Table 1.

In qualitative studies, sample sizes are generally determined according to the rule of saturation/information redundancy and are often in the range from 20 to 50–60 participants [38]. A focus group study, as a type of qualitative study, generally also follows the saturation rule. As for the number of groups, Guest et al. (2017) established in a systematic analysis of focus group studies that 90% of the topics were discoverable within three to six focus groups, while three focus groups were also enough to identify all of the most prevalent themes within the data set [39].

To assure sincere participation, given the utmost sensitivity of the topic, the relatively small size of the Croatian medical community, and the moment at which the study was conducted, we decided not to collect any additional demographical data from the participants. MRQual, a web-based platform for conducting focus groups was used as a data-collection tool. The participation was anonymous, i.e., the participants were able to freely choose anonymous usernames and to change or delete their contributions. More specifically, the moderators were only familiar with the information which participants had registered for participation and thus were only able to assign the participants to the appropriate group. Each focus group lasted for seven days. No incentives were offered for participation. The contributions were based on a focus group guide containing questions that directed the discussion and were structured around specific discussion topics. Namely, the participants were expected to state their opinions and experiences related to vaccination and to give their feedback on the comments left by the other participants and the moderator if they wanted to do so. The focus group discussion topics are shown in Table 2.

The study fully considered possible ethical dilemmas, having in mind the sensitivity of the research topic, as well as the nature of qualitative research and its general sensitivity to ethical issues. As a consequence, the study was conducted by choosing the data collection method that fully guarantees the anonymity of the participants (asynchronous online focus groups). The Ethics Committee of the Faculty of Humanities and Social Sciences Josip Juraj Strossmayer University of Osijek approved the study (Ethical approval code: 2158-83-07-20-3) and informed consent was obtained from each participant.

As a method of data analysis, thematic analysis was applied. We applied the framework of analysis proposed by Braun and Clarke, and followed suggested steps (familiarizing with data, generating initial codes, recognizing then reviewing and naming emerged themes, producing the report) in reading and analyzing transcripts of conducted focus groups [40]. All of the identified emerging themes are thoroughly described in the following section, along with the extracts from the transcript which are most illustrative for the specific theme. To gain deeper insights into the unstructured data, as well as to better manage and visualize the emerging codes, we used the latest version (Release 1.0) of NVivo, a qualitative data analysis software package. We also provide a thick description and concrete details by providing verbatim transcriptions of the focus groups participants. However, given the anonymity of the study, we only list participants’ study identification numbers and the study identification number of the focus group. In other words, we provide no further participants’ demographics, other than the healthcare level in which they work that is visible from their membership in the focus group. The purposive nature of the sampling procedure guarantees multivocality as well. We hope to find resonance among the study readers by providing meaningful details, as well as naturalistic generalizations and transferable findings. Triangulation of the study results is planned in the next phases of the project (quantitative research). Overall, given that the paper presents the results of qualitative research, we aimed to satisfy the eight key markers of quality in qualitative research developed by Tracy [41]: worthy topic, rich rigor, sincerity, credibility, resonance, significant contribution, ethics, and meaningful coherence.

## 3. Results

### 3.1. Overall Attitudes towards Vaccination 

As probably could have been expected, the participants generally expressed positive attitudes about vaccination, which include the understanding of the vaccines as a great scientific and human achievement, the acknowledgment of the efficacy of the vaccines, and expressing confidence in the safety of the vaccines:

e.g., “I absolutely support vaccination. In addition to getting vaccinated regularly against the flu, I also vaccinated my children against pneumococcus, when that vaccine was not on the Vaccination Calendar. I also support HPV vaccination.” (P24, FG2).

e.g., “I have no dilemmas about the safety and effectiveness of the vaccine. I believe that vaccines can cause some harmful effects in the body, but I think they are mostly short-lived, transient, and mild.” (P4, FG1).

e.g., “As a long-term vaccination provider, I believe that the benefits of vaccination far outweigh the potential risks. Vaccines are safe, and side effects, in particular, the more serious ones are extremely rare.” (P8, FG1).

Slightly skeptical views about vaccines were also noticeable. Concerns about the research ethics and the various phases of vaccine production were expressed by some participants, and such concerns often overlap with the premise that not all vaccines are equally safe or appropriate for all patients and population segments. In addition, some skepticism was also expressed about new vaccines in the context of accelerated vaccine production.

e.g., “I don’t think all vaccines are equally safe. This depends on the clinical research conducted: a good randomized and comprehensive study at all ages of the population is needed, and a record of all side effects.” (P20, FG2).

e.g., “I have an affirmative attitude towards vaccination but only in the case of traditional vaccines that have passed all the necessary administrative and scientific legislation. I am relatively reluctant to the idea of seasonally vaccinating the general population against influenza, except when it comes to at-risk individuals, due to old age or comorbidities that could potentially lead to a fatal outcome in the event of infection.” (P38, FG3).

e.g., “I have the impression that at one time the incidence of autism in children of approximately the same age (back 10-15 years) was higher in the population in which I move, and on several occasions, I watched TV shows on the subject and read articles on the Internet portals, but I cannot generally take a stand about it because I haven’t covered the topic in enough detail. And I repeat, it’s just my impression.” (P26, FG2).

**Figure 1:**
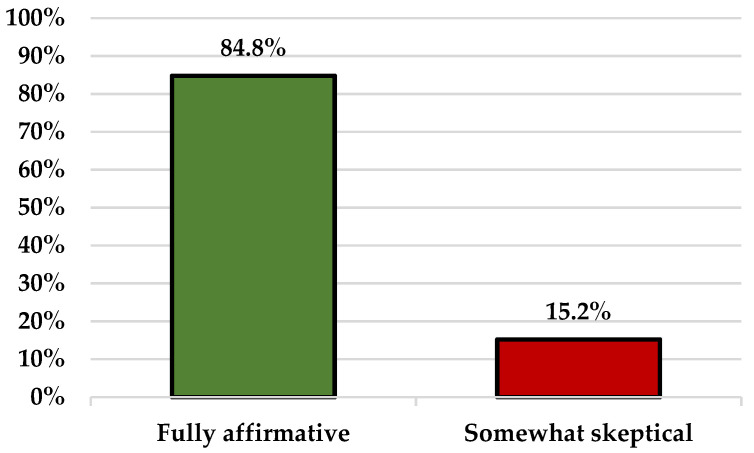
The overall attitudes towards vaccination.

Quantitatively speaking, 39 (84.8%) participants expressed a fully affirmative view towards vaccination, while 7 (15.2%) participants gave a mixed answer by expressing some skepticism about the process (Figure 1). However, we need to emphasize here that the numbers provided here and in the rest of the analysis are given only for descriptive and illustrative purposes since the qualitative nature of this study and its limited sample do not justify more precise estimates and statistical generalizations.

### 3.2. Mandatory Immunization Program

All participants agreed that the vaccination program should remain mandatory. They see the mandatory vaccination program as being in line with scientific development, and that the educational programs provided by the government can emphasize the importance of mandatory vaccination.

e.g., “I think the program is changing in line with the development of scientific knowledge. It is important for parents to plan and apply for vaccinations on time. I believe that vaccination according to the calendar should still be mandatory. I would add the chickenpox and rotavirus vaccine to the vaccination calendar.” (P2, FG1).

Some differentiation of answers about mandatory vaccination is noticeable by some participants highlighting exceptional cases in which refusal of mandatory vaccination can be acceptable. Such cases mainly relate to the health conditions that would make it impossible to receive the vaccine.

e.g., “Worldview reasons (attitudes about health, personal freedoms and the state, religion, etc.) are by no means acceptable as a basis for exceptions. Medical reasons and contraindications are definitely justified.” (P11, FG1).

Some participants expressed the need for a social response to those who unjustifiably refuse vaccines, mostly by coercive sanctions. According to some participants, the social reaction should represent a response to a refusal to fulfill obligations shared by the community members. Legal reactions are mainly focused on financial sanctions in case of an illness that would have not occurred if the vaccines had been applied.

e.g., “Nobody leaves it to the citizens to take care for a technical inspection if they want to, and if they don’t, they don’t have to. They are forced to do so if they want to drive among other traffic participants, so I think that if someone wants to be a full member of the community, he/she has to follow the rules of the vaccination program.” (P41, FG3).

e.g., “In Croatia, the courts of law punish too slowly (as customary in our country) and with minimal penalties vaccination refusal within the compulsory vaccination calendar which sends a devastating message when it comes to the health protection of citizens.” (P42, FG3).

On the other hand, one of the highlighted problems in the context of the vaccine implementation was vaccine shortages, which might deter some people from deciding to vaccinate.

e.g., “There are usually some problems with the flu vaccine shortages every year, in terms of ordering and the final number of doses to be received.” (P2, FG1).

e.g., “We don’t always have enough supplies. The authorities do not care how we feel because we are the ones who suffer the frustration of patients as we explain why there is no vaccine. Therefore, we lose the motivation of the patients. And with the compulsory vaccines, the shortage is always a good excuse for parents not to vaccinate their children.” (P45, FG3).

e.g., “It happens occasionally that there is a shortage of some vaccines, but it is a very short period. Sometimes it causes us some inconvenience, but we somehow make up for it, we manage, we borrow the vaccines from a colleague, we re-schedule the patients.” (P31, FG2).

When we analyzed opinions about MIP all participants, 46 (100.0%), stated that vaccination program should remain mandatory in Croatia. 

### 3.3. Vaccination Side-Effects

When it comes to reporting side-effects, the participants unanimously stated that not all side-effects are being reported, because the minor and transient effects are not perceived as a danger. Several participants also added that the real side-effects are not the cause that makes patients vaccine-hesitant.

e.g., “I know that not all side-effects are being reported. Insignificant side effects that have passed, mild nausea, mild headaches, local pain, etc. Too much bureaucracy for something that has passed.” (P40, FG3).

e.g., “Since I have vaccinated thousands of children, I can claim that the side effects are rare. Not all expected mild side effects are reported for sure because when you warn your parents about them in advance, they do not even contact a doctor. After all, they know how to take care of them themselves.” (P44, FG3).

e.g., “Vaccine side effects are uncommon, most commonly local, and very mild. Side effects are not a reason which discourages patients from vaccination. The parents themselves say that they did not notice anything about the vaccination, but here they are –afraid because they have heard and read…” (P1, FG1).

During the quantitative exploration of participants’ opinions regarding the report of vaccine side-effects, all participants 46 (100.0%), agreed that although vaccine side-effects should be reported the minor and transient side-effects are usually not reported.

### 3.4. Communication with Vaccine-Hesitant Patients

As already mentioned, all participants agreed that the vaccination program should remain mandatory. However, there is some disagreement over the issue of the possibility to communicate with the vaccine-hesitant persons and to change their opinions and decisions, wherein most of the participants expressed a pessimistic outlook about the communication outcomes. Additionally, most participants professed the ability to differentiate between various types of vaccine-hesitant persons.

e.g., “We do not have enough time to communicate with the patients and to reason with them. I think that there are too many expectations from us and that we cannot handle the situation. Other specialists, such as psychologists, should probably be more involved.” (P6, FG1).

e.g., “I have to admit that entering into a discussion with hesitant parents often leads to verbal conflict and that these parents are quite aggressive in defending their theories. What I also noticed is that these are often people of better financial status and that they brag with comments like—If needed, my child won’t go to kindergarten, or he/she will go to a private school! I don’t mind paying 100 fines if I have to!” (P25, FG2).

e.g., “From my experience when I was in primary care, people don’t know how the vaccine works. I always drew the mechanisms, as I would do to children. I didn’t get into conflicts with them; I quickly judged who didn’t want to hear me. I do listen to them, but I have to do my job” (P37, FG3).

e.g., “Everyone who does not want to vaccinate his/her child ends up with an epidemiologist. Parents come to the interview in advance because they have to do it without the desire for actual conversation, with the already prepared common story of whether I will sign that nothing will happen to the child. About five minutes, sometimes less, is enough for such parents. Of the hundreds of conversations, only one parent told me after the interview that they would vaccinate their child. If forced to do so because of the kindergarten, parents agree to one dose of any vaccine because they receive a confirmation from the pediatrician that they are covered by regular vaccinations, and once they enroll the child they stop vaccinating.” (P43, FG3).

e.g., “It usually happens that a whole group of parents “follows” one to two loud people who are against vaccination. Given the type of work I do, I talk about vaccinations quite often with parents, but also with their children, and one part of them changes their mind (positively) after talking and answering their questions.” (P7, FG1).

e.g., “There are four types of non-vaccinating parents. The first type is anti-vaxxers, they come with documents, forms, ask you to sign something, send letters and threaten a lawsuit through law firms, are aggressive in their performance, have an opinion about vaccination based either on conspiracy theories or on some bad experience. The second type comprises parents who will never say they refuse to vaccinate, but do not respond to written calls for vaccinations. They always say that the child just now has a cold or cough or fever. The third one is the ones who do not care about vaccination because they do not care about their children in general. They are already under the surveillance of social services. The fourth type is parents who have concerns about vaccination, especially if there is something new in the calendar or some reaction to the vaccine has occurred. They will clearly express their problem or doubt. It is worth talking to them and dedicating time to them.” (P38, FG3).

**Figure 2:**
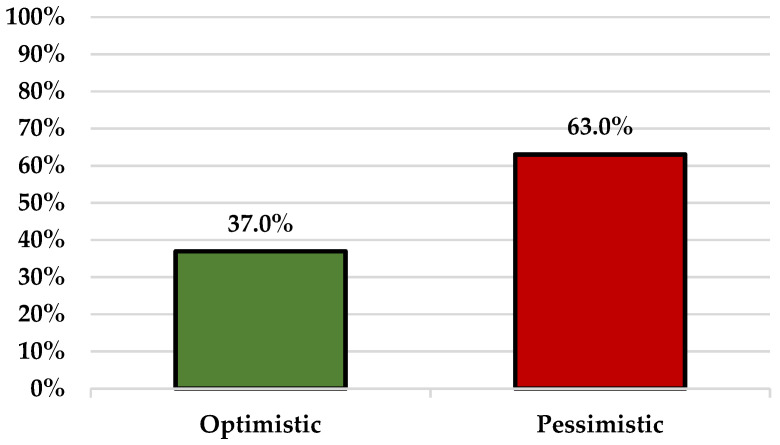
Communication with vaccine-hesitant patients.

Regarding the communication with the hesitant patients, 29 (63.0%) participants expressed a pessimistic outlook about the possibility of changing the already established attitude of the skeptical patients, while the remaining 17 (37.0%) participants had a mainly positive outlook (Figure 2).

### 3.5. COVID-19 Vaccination

Opinions on COVID-19 vary from those being completely supportive to those who are somewhat suspicious. The reasons for getting vaccinated are related to the general trust in the science and healthcare system. Suspicion and reluctance mainly stem from the skepticism about the rapid development of such vaccines, and from a perceived need to differentiate between various population segments (such as those based on age and health status).

e.g., “I am absolutely in favor of getting vaccinated against COVID-19 infection, and I plan to get vaccinated against it, as do most other colleagues in my work environment. I believe that if science and relevant institutions have given the “green light” to a particular vaccine, we should believe it, and not question the intentions and support various conspiracy theories.” (P40, FG3).

e.g., “I am generally a supporter of vaccination, including this one against COVID-19. I plan to get vaccinated, as does my mom and husband, but I don’t plan on vaccinating my children. I also plan to recommend my patients get vaccinated against this virus on the same principles as stated above.” (P22, FG2).

e.g., “I will not vaccinate against COVID-19 immediately. The pressure and the race to produce a vaccine at all costs will take its toll.” (P9, FG1).

e.g., “Regarding the upcoming vaccines against COVID-19, I do not plan to be vaccinated immediately, because I believe that too short a period has passed from the beginning of vaccine development to its use for the general population.” (P14, FG3).

Some participants think COVID-19 vaccination should be in the same category as flu vaccines, meaning they shouldn’t be mandatory and should firstly be provided to those who are at high risk for the potential of serious disease complications.

e.g., “For now, I would not prescribe mandatory vaccination against COVID-19. I would put it in the flu vaccination category, which is seasonal and not mandatory.” (P23, FG2).

e.g., “Obligations create resistance. I would equate COVID-19 vaccine with the flu shot with a recommendation for health facilities and homes for elderly.” (P39, FG3).

**Figure 3:**
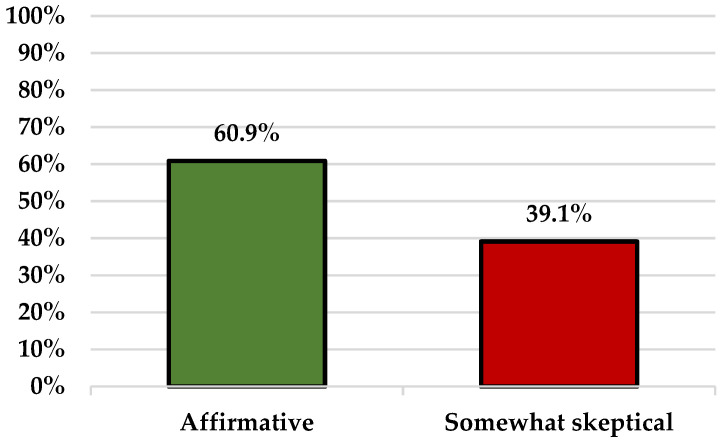
COVID-19 vaccination.

Overall, the number of participants without any doubts related to the COVID-19 vaccines is higher (28; 60.9%) than the number of those who expressed some skepticism (18; 39.1%) (Figure 3).

## 4. Discussion

The results of this qualitative study confirmed the existence of some of the previously detected reasons for vaccine hesitancy among HCWs. The participants expressed a high level of support for Croatian MIP and vaccines in general. However, some skepticism was conveyed regarding new vaccines and the regulatory processes of their approval. Some participants also reported the possible negative effects of the vaccine shortages, although they are quite rare. The results are similar to the study from Bosnia and Herzegovina [42] and to the study conducted in Croatia that showed that some Croatian HCWs do express vaccine hesitancy [12]. This is of particular concern because a previous study showed that the attitude and knowledge of HCWs about vaccines can influence their intention to recommend vaccination to their patients [15,43,44,45,46]. Consequently, it is highly important that public health leaders better understand HCWs’ vaccine-related behaviors and attitudes and take adequate steps to counter the hesitancy. Even though the results of qualitative studies cannot lead to reliable generalizations, we can note that a significant number of participants raised concerns over the approval of COVID-19 vaccines, especially given their rapid development. New vaccines, such as the COVID-19 vaccines, were singled out due to a perceived lack of testing for vaccine safety and efficacy. This confirms results of studies conducted before the COVID-19 pandemic which also showed HCWs concerns about new vaccines [47,48], as well as the results of studies that revealed that concerns with the speed and approval of the vaccines as a strong predictor of HCWs COVID-19 vaccine hesitancy [49,50,51].

However, this study also revealed some new characteristics of vaccination behavior among HCWs, which can be tentatively explained by its embeddedness in the social context in which vaccination takes place. The most interesting and novel finding of this study is related to the communicative aspect of the vaccination process and its possible connection with a social roles conflict that arises from the contradictory pressures to which medical doctors are exposed. Namely, even though some participants in the current study showed a good understanding of the need to effectively communicate with their patients by using personal stories and other easily understandable communication devices [52], it is interesting to note that several participants already have the firmly established and very elaborated a priori classifications of vaccine-hesitant persons, so that they can allocate their time and communication efforts accordingly, to be able to work more efficiently and not to waste time on the probably unsuccessful trust-building communication encounters. Using Emanuel and Emanuel’s classification of physician–patient relationship models, we can note that some participants do not use the interpretive model (elucidating and interpreting relevant patient values as well as informing the patient and implementing the patient’s selected intervention). Instead, they often fall back on either informative (providing relevant factual information and implementing patient’s selected intervention) or paternalistic model (promoting the patient’s well-being independent of the patient’s current preferences) [53]. This finding is similar to the study of Finnish HCWs which reported that around 13.8% of HCWs do not actively guide the patient in any direction when the hesitancy concerns the childhood vaccines [44]. In a similar vein, a study using the American Academy of Pediatrics Periodic Surveys from 2006 and 2013 revealed that in 2013 around 11.7% of the pediatricians reported “always” dismissing patients for continued vaccine refusal, which is a significant increase from 6.1% in 2006 [54]. The effectiveness of various communication styles in increasing coverage rates is still a debated issue. Even though the more patient-orientated styles are often recommended [55,56,57]; some studies demonstrated that a more provider-centered approach or an approach with a presumptive initiation of communication could be more efficient [58,59]. Be that is it may, a recommending behavior is always related to vaccination success regardless of the communication style that providers use [60].

The fact that some Croatian HCWs do not effectively communicate with the vaccine-hesitant patients may be a point of concern because it has been reported that a lack of commitment from health professionals is one of the major reasons for refusing vaccines [44,61,62,63,64]. Some studies revealed that the reason for such behavior may be inadequate communication skills which are required to respond to parental hesitancy or vaccine refusal [42,65]. The latter points to the conclusion that some type of communication training would be of use to the HCWs and might enable them to efficiently approach even the extremely vaccine-hesitant patients. However, the communication breakdowns with patients demonstrated in our study can also be explained by the contradictions that are embedded in the roles of “good doctors” and “good patients”. As Deml et al. elaborate [66], some deep-rooted paradoxes stem from the contradictory expectations which doctors and patients face when they engage in mutual communication. The doctors are expected to be good listeners and to respect patients’ rights and autonomy, while at the same time they should follow the strict vaccination schedules and programmed coverages. Namely, there is a deep paradox in being required to respect the patient’s autonomy and being obliged to achieve immunization coverage rates as high as possible in as short a time as possible. It is not surprising that often MDs do not use patient-centered communication approaches in these situations, since such approaches are economized with and thus more likely to be found where severe health conditions with numerous physical symptoms are diagnosed [67]. However, patients are expected to be autonomous individuals that make their own choices, especially in the wider social processes that favor individualism and consumerism in all social areas, healthcare included. More often than not, vaccine-hesitant persons are the ones who gather information and feel the internal pressure for making a responsible and informed decision dictated by the consumeristic health philosophy [68]. Therefore, we might also conclude that for the above-mentioned reasons both doctors and patients are given a “false autonomy” when making vaccination decisions, thus leading to numerous communication breakdowns and essentially empty encounters. Encounters of that kind are likely to be damaging bearing in mind that the very act of attentive listening not only helps with data gathering and diagnosis but also strengthens the doctor-patient relationship [69,70,71]. In other words, we posit that the examples of unsuccessful doctor-patient communication in the vaccination context may result from the dual and contradictory pressures stemming from the job demands and the demands coming from the professional roles and the consumeristic and patient-centered contemporary healthcare systems. In the light of the above-mentioned social constraints, maybe it is not surprising that, in a rare instance of a randomized trial in this field of study, Henrikson et al. [72] did not find any effect of communication training of MDs on vaccine hesitancy of their patients. 

The declining general trust in the medical profession places an additional burden on the communication process. Wolfensberger and Wrigley make a convincing case by emphasizing the importance of the following five reasons for the decline of trust in MDs: (1) the discrediting of professionalism, (2) the insistence (and difficulty of) assessing physicians’ trustworthiness, (3) the disavowal of the basic tenets of scientific medicine, (4) the commodification of medicine and the re-conceptualization of physicians as dependent employees, and (5) changes of risk perception and risk acceptance. The medical profession lost its professional authority, thus status-based trust is exchanged for merit-based trust, which is notoriously difficult to assess. The disavowal of the basic tenets of scientific medicine refers to the postmodern cultural ambiance which questions “scientific monopoly” on truth by emphasizing relativism. The commodification of medicine undermines trust by framing the relationship between physicians and patients as a business relationship, which implies that physicians are not committed to patients because they believe that is the right thing to do (moral obligation), but because they will be paid for (monetary motivation/self-interest). Finally, as Wolfensberger and Wrigley note, even though the standard of living is rising and humans face fewer risks than before, given their visibility due to the abundance of information, risks are believed to have increased [73]. Additionally, most risks in contemporary societies are manufactured, i.e., they are produced by the human agency [74,75,76,77]. This also led to the existence of competing knowledge systems, some of them being non-expert ones, which further undermine trust in scientific knowledge. Without external support of trust, several participants from our study expressed their choice to economize with the available time and not to fight “lost battles” [78]. As noted above, the communication breakdowns might be the result of deficient communication skills, but they can also be the consequence of the social roles that are deeply embedded in the wider social and cultural processes which cannot be easily managed and changed. Therefore, vaccine hesitancy research has to include wider topics of medical decision making, trust, as well as different ways of risk conceptualization and management [79].

Overall, in this paper, we tried to provide significant theoretical and empirical contributions by integrating vaccine hesitancy research with studies concerning the social context of healthcare systems, which is rarely the case. A systematic literature review of the research that covered vaccine hesitancy among HCWs showed that the studies are mainly focused on the connection between vaccination hesitancy and recommending behavior, while the communication processes between HCWs and their patients are very rare [15]. Therefore, our study extends previous research by emphasizing the importance of communication processes and their connection with wider social processes. In other words, we posit that particular characteristics of contemporary “risk societies” and healthcare systems, such as increased risk perceptions, commodification and commercialization of health, and medical workers’ social roles should be taken into account in vaccine hesitancy research, especially when it comes to the studies undertaken to explain vaccination attitudes of HCWs and their relation to the success or failure of immunization programs. Our conclusions interconnect various research findings within the study, as well as provide a possibility for extending vaccine hesitancy research in new directions. Therefore, we believe that the results of our study could be heuristically useful for providing a ground for future studies dealing with more specific issues within the area of vaccination attitudes among the HCWs in Croatia and other countries. However, we need to emphasize that our main findings and the proposed theoretical explanations need to be tested in future quantitative research. A qualitative study, such as this one, cannot bring precise quantitative assessments and reliably establish causal mechanisms that can explain empirical phenomena.

Our study is not without limitations. Even though, as in all qualitative studies, the question of the sample representativeness and the possibility of reliable generalizability of the study results can be rightfully raised, this study was successful in bringing some new and important insights into Croatian MDs’ attitudes and expectations regarding vaccination amidst COVID-19 pandemic. As for the method of data collection, asynchronous online focus groups did offer significant practical and methodological advantages in our research situation, but they also provide only limited group interaction and consequently fewer rich results in comparison with face-to-face focus groups. In addition, there is also a concern that some participants may have given the socially desirable answers although they had been given full anonymity assurance. With all aforementioned limitations, further confirmation of the study’s importance and value lays in new research directions that have been revealed. Namely, after detecting an interconnection between HCWs’ vaccination attitudes and their recommendation behavior, in future studies, it would be important to evaluate this interconnection in more detail and to further investigate why some HCWs are not successful in directing their patients to immunization decisions. 

## 5. Conclusions

A qualitative study is judged by the novel insights that it brings to the surface. In our opinion, this study brought about some important insights into the vaccination attitudes and experiences of medical doctors and their views about communication encounters with patients, which have not been thoroughly studied previously. As the main conclusion, we posit that MDs find themselves in a delicate situation where a fine balance between time-consuming communication with patients and the demands for maintaining satisfying vaccination uptake is of vital importance, which leads to the elaborate categorizations of patients and economizing with time and effort which are invested in communication encounters. The situation arises from the social roles conflict, which is embedded in wider social values and expectations, especially within the social context of the declining trust in the medical profession and science in general. Consequently, our study suggests that the communication problems do not arise in the doctor’s office, and therefore cannot be solved without addressing the social forces that cause trust deficiencies. Nevertheless, there is a need to persuade MDs and all HCWs who participate in the vaccination process to systematically and reflexively explore and possibly revise the aforementioned a priori categorizations of patients. Legitimate concerns of patients need to be addressed, especially given that vaccine hesitancy is a dynamic phenomenon, with some new possible factors entering the field. The lack of interest in communication that follows from the swift generalizations naturally leads to the psychological reactance on the patients’ side and communication breakdowns, thus failing to achieve the desired public health outcomes.

## Figures and Tables

**Table 1:** The focus group characteristics.

Focus Group Characteristics	Focus Group 1 (FG1) N = 18	Focus Group 2 (FG2) N = 17	Focus Group 3 (FG3) N = 11
Age (years) Mean ± SD *	44.3 ± 7.7	45.9 ± 5.5	42.8 ± 9.7
Gender Male:Female	5:13	8:9	5:6
Participants job description	primary care pediatricians, general practitioners	secondary care pediatricians, other medical specialists	epidemiologists, other public health specialists
Participants institution	healthcare centers	general and county hospitals, clinical hospitals, and clinical hospital centers	county public health institutes and Croatian Institute of Public Health

* SD—standard deviation.

**Table 2:** The focus group discussion topics.

Topic Number	Topic Content
1	Overall attitudes towards vaccination
2	Mandatory immunization program
3	Vaccination side-effects
4	Communication with vaccine-hesitant patients
5	COVID-19 vaccination

## Data Availability

Not applicable.
